# Clonal Analysis in Mice Underlines the Importance of Rhombomeric Boundaries in Cell Movement Restriction during Hindbrain Segmentation

**DOI:** 10.1371/journal.pone.0010112

**Published:** 2010-04-12

**Authors:** Eva Jimenez-Guri, Frederic Udina, Jean-François Colas, James Sharpe, Laura Padrón-Barthe, Miguel Torres, Cristina Pujades

**Affiliations:** 1 Department of Experimental and Health Sciences, Universitat Pompeu Fabra, Barcelona, Spain; 2 Parc de Recerca Biomèdica de Barcelona (PRBB), Barcelona, Spain; 3 Department of Economics and Business, Universitat Pompeu Fabra, Barcelona, Spain; 4 European Molecular Biology Laboratory-Centre for Genomic Regulation (EMBL-CRG) Systems Biology Research Unit, Centre for Genomic Regulation, Barcelona, Spain; 5 EMBL-CRG Systems Biology Research Unit, Institució Catalana de Recerca i Estudis Avançats (ICREA), Centre for Genomic Regulation, Barcelona, Spain; 6 CNIC, Madrid, Spain; National University of Singapore, Singapore

## Abstract

**Background:**

Boundaries that prevent cell movement allow groups of cells to maintain their identity and follow independent developmental trajectories without the need for ongoing instructive signals from surrounding tissues. This is the case of vertebrate rhombomeric boundaries. Analysis in the developing chick hindbrain provided the first evidence that rhombomeres are units of cell lineage. The appearance of morphologically visible rhombomeres requires the segment restricted expression of a series of transcription factors, which position the boundaries and prefigure where morphological boundaries will be established. When the boundaries are established, when the cells are committed to a particular rhombomere and how they are organized within the hindbrain are important questions to our understanding of developmental regionalization.

**Methodology/Principal Findings:**

Sophisticated experimental tools with high-resolution analysis have allowed us to explore cell lineage restriction within the hindbrain in mouse embryos. This novel strategy is based on knock-in alleles of ubiquitous expression and allows unrestricted clonal analysis of cell lineage from the two-cell stage to the adult mouse. Combining this analysis with statistical and mathematical tools we show that there is lineage compartmentalization along the anteroposterior axis from very early stages of mouse embryonic development.

**Conclusions:**

Our results show that the compartment border coincides with the morphological boundary in the mouse hindbrain. The restriction of the cells to cross rhombomeric boundaries seen in chick is also observed in mouse. We show that the rhombomeric boundaries themselves are involved in cell movement restriction, although an underlying pre-pattern during early embryonic development might influence the way that cell populations organize.

## Introduction

Compartments were originally described in *Drosophila* imaginal discs as subdivisions of organ primordia occurring on an homogeneous epithelial cell field and whose coherence is maintained by cell lineage [1]–[3]. Compartment boundaries are unique lines at stereotyped positions in a developing organ, across which cell intermingling does not take place. *Drosophila* compartmental organization is a background subdivision of embryonic fields that serves to establish positional references in the primordium but is not necessarily related to anatomical boundaries in the organism.

Lineage restriction units resembling *Drosophila* compartments have also been described in vertebrates, such as rhombomeres (r) in the hindbrain. These are the result of a segmentation process along the antero-posterior (AP) axis of the neural tube. Pairs of rhombomeres cooperate to generate a metameric organization that underlies the repeating sequences of cranial branchiomotor nerves [4]. This transitory rhombomeric organization is also critical for segmental specification and migration of neurogenic and branchial neural crest cells [5]. The appearance of morphologically visible rhombomeres is a dynamic process that requires the segment restricted expression of a series of transcription factors, which position the molecular rhombomeric boundaries, followed by the establishment of morphological boundaries [6]. The matching of the rhombomere pattern with an underlying cellular organization and gene expression pattern indicates that segmentation is important in the construction of the hindbrain. Studies of cell commitment and gene expression suggest that the subdivision of the hindbrain into segments and the specification of the AP identity emerge from a prepattern in the early neural plate [6].

Most lineage restriction borders described in both vertebrates and insects are associated with signalling centres [7]. This suggests that a major role of lineage compartments during embryonic development is signalling-centre stabilization. In contrast to *Drosophila* compartments, however, all lineage restrictions described so far in vertebrates coincide with, or anticipate, anatomical or cell-type discontinuities. The known restrictions in vertebrates may thus not be a background subdivision of embryonic fields, but might instead largely correlate with strategies to allocate cells fated to different anatomical structures.

Some of the questions that have challenged developmental biologists in the last years are when and how rhombomeric boundaries are established and subsequently maintained. Pioneering work in the chick hindbrain, involving labelling of multiple neuroepithelial cells with a lipophilic dye, identified cell lineage restriction boundaries at the borders between rhombomeres [8]. From this work, the authors concluded that individual cells were initially capable of considerable movement within the sheet of the neural epithelium; however, cells did not freely move anymore after the establishment of the rhombomeric boundaries occurred. This restriction of cell migration is thought to be required for each segment to maintain a specific pattern of gene expression and thus a distinct AP identity [9].

To investigate the cell behaviour during lineage restriction, we have undertaken a high-resolution clonal analysis in the hindbrain of mouse embryos. This novel strategy is based exclusively on knock-in alleles of ubiquitous expression and allows unrestricted clonal analysis of cell lineage from the two-cell stage to the adult mouse [10]. Using this strategy, we have analyzed the cell clone distribution in the developing mammalian hindbrain. Combining this analysis with statistical and mathematical tools we demonstrate that there is lineage compartmentalization along the AP axis. This is observed from very early stages of embryonic development (E5.5), indicating that patterning along this axis might involve restrictions of cell dispersion at specific axial positions. Our results show that the compartment border coincides with the morphological boundary and reinforces the importance of the rhombomeric boundaries themselves for the cell movement restriction to different rhombomeres.

## Results and Discussion

Our aim was to clonally label cells in the mouse embryo to explore cell lineage restriction in the mammalian hindbrain. Rhombomeric boundaries act as borders of cell restriction in the chick embryo [8]. We performed experiments and statistical analysis to complement the existing information with a mammalian model. Our data confirmed the same restrictive pattern is found in the mouse embryo, and reinforces the role of rhombomeric boundaries as restrictive borders to cell movement very early during embryonic development.

### Characterization of an inducible system for clonal analysis in the hindbrain of mouse embryos

To perform systematic clonal analysis in the developing mouse embryo, we applied a method that relies on the use of ubiquitously expressed knock-in alleles and is suitable for non-invasive permanent cell labelling during embryonic development [10]. This genetic strategy is based on the site-specific induction of Cre recombinase. Cre-mediated recombination is monitored by expression from either the recombination-activatable *R26R* or *R26R-EYFP* knock-in alleles [11], [12]. We used conditions for low frequency recombination, such that when a group of positive cells is detected, the probability of polyclonal origin is low [10]. Once Cre-mediated recombination is induced upon tamoxifen (TM) administration, cells are genetically marked, therefore labelling does not dilute with cell division and growth. This allowed us to follow descendants after as many cell divisions as desired. Marking cells genetically allows an alternative approach to labelling with a dye as done previously in chick embryos.

**Figure 1:**
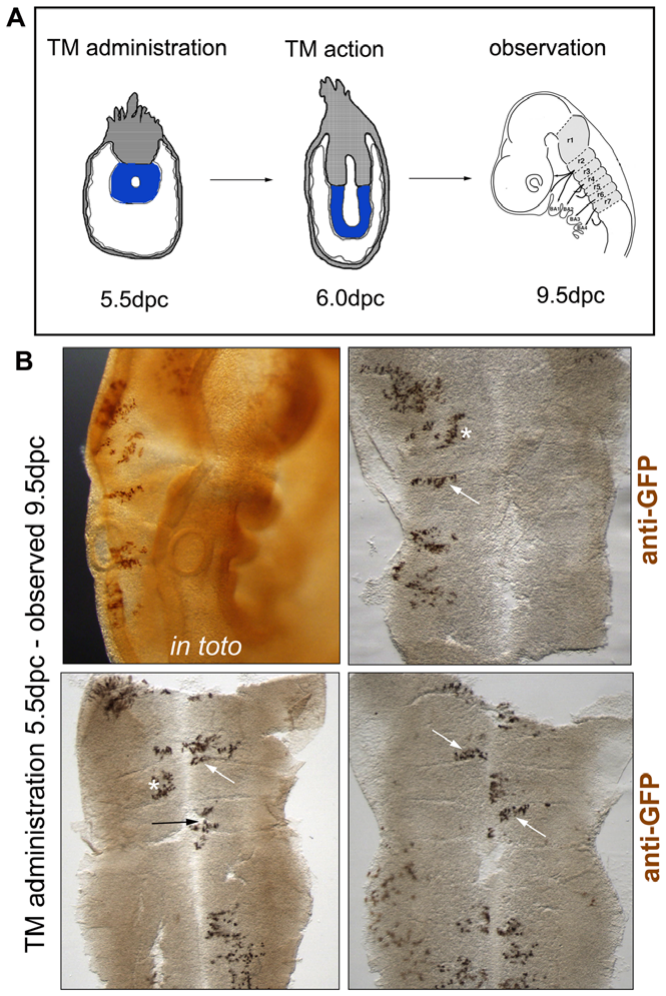
Clones respect the rhombomeric boundaries upon early labelling. (A) Scheme depicting the embryonic stage upon TM administration (5.5 dpc), the embryonic stage of Cre-induction (6.0 dpc), and the stage when the observation was carried out and the hindbrain dissected (9.5 dpc). Note the dramatic change in embryo morphology between the time of TM administration and the time of embryo observation. Blue areas in 5.5–6.5 dpc depict the embryonic tissue. r1-r7, rhombomere 1–7. (B) Immunostaining to reveal GFP-positive clones in whole embryos (*in toto*) and in flat-mounted hindbrains.

We induced recombination at stages ranking between inner cell mass stage (5.5 dpc) and 13-18 somites stage (8.5 dpc) that is, before and during the early establishment of the rhombomere boundaries. For this purpose, mice pregnant at stages from 5.5 dpc to 8.5 dpc were injected with TM, and the distribution of induced clones examined once the hindbrain boundaries are morphologically visible (Table 1). There is a delay between TM administration and induction of the recombination, which has been estimated between 8 h and 12 h ([10]; Figure 1A and data not shown). Analysis of positive cell distribution within the embryo was performed by direct whole-mount visualization of *LacZ* or GFP staining. GFP signal is too weak to perform direct observation of endogenous fluorescence, hence the need for immunostainging to visualise GFP expression. We pooled litters into experimental groups according to the TM administration time and the moment of observation (Table 1). For each experimental group of embryos, we calculated: i) the frequency of induction (and therefore recombination) within the group (number of embryos with recombination events/total number of embryos); and ii) the frequency of induction within the hindbrain from r2 to r6 (number of embryos with recombination events in the hindbrain/number of embryos). Table 1 shows data from the different experimental groups. Although the quantity of TM administrated was comparable in all females, the output of recombination was very heterogeneous (Table 1, data not shown), suggesting that either the activity of the TM was very variable, or that the delivery of the TM to the embryos changed among females. Since it was very difficult to assess the real TM activity received by the embryos, we administrated a TM dose that let us be confident in the maximum number of events, but not in the minimum number. Individual clones within the hindbrain contained between 10 and 60 cells and many of them extended up to 80% of the rostrocaudal length of a rhombomere. However, in three cases the clones extended the whole rhombomere AP length. The clones of labelled cells were quite compact although they could be mixed with unlabelled cells in a ratio 1∶0.25 for the most compact clones and in a ratio of 1∶5 the most widely dispersed clones. This intermingling with unlabelled cells showed that individual cells were capable of cell movement within the sheet of the neural epithelium, although this was quite restricted observing the low clone dispersion. In chick, neuroepithelial cells take around 8 h to proceed along the cell cycle [13], meanwhile in mice, cell cycle length is approximately of 10 h. Therefore, as expected, the size of the induced clones was proportional to the time between induction and observation (data not shown). When recombination was induced at 5.5 dpc, individual clones observed at 9.5 dpc originated most probably from a same progenitor cell, whose descendants had contributed to different embryo lineages and could be found in all embryo layers. In that case, although individual observed clones were not strictly originated from independent recombination events, they could be considered as independent clones in terms of cell behaviour.

**Table 1:** Relative frequencies of induction (and therefore recombination).

TM administration	Observation	n	F	HB F
5.5 dpc	9.5 dpc	128	0.5391	0.2422
5.5 dpc	10.5 dpc	106	0.1981	0.0849
6.5 dpc	10.5 dpc	23	0.6522	0.1739
7.5 dpc	10.5 dpc	83	0.9518	0.3976
8.5 dpc	10.5 dpc	63	0.9365	0.3175

n: number of embryos.

F: total relative frequency.

HB F: frequency within the hindbrain.

Pregnant mothers were administrated with TM at different developmental stages. Embryonic development proceeds until the desired observation stage. n, number of studied embryos; F, total relative frequency (*x/y*) where *x* is the number of embryos displaying recombination events, and *y* the total number of embryos per experimental group; HB F, relative frequency within the hindbrain.

The use of this method gets rid of the dilution of the signal that can, and mostly does, occur when labelling cells with dyes. In this sense, this is a better method for cell tracking than the use of dyes, and has already been used for other purposes such as studies of cell compartmentalisation within the limb [10]. Nevertheless, one limitation of the system for our purpose is that it cannot target specific regions and cannot be tested at different times in the very same embryo. Increasing the number of collected embryos will give more chances of observing the clones in the desired regions. Hence, this would be, as we have done, the best and easiest method to overcome that problem. We also think the method might be improved if life tracing in the embryos could be performed, and discuss this issue in the next section of this Results and Discussion.

### Clones respect the rhombomeric boundaries upon early labelling

We examined the spatial distribution of clones in relation to morphological rhombomeric boundaries when cell labelling takes place before boundaries are established (5.5 dpc–7.5 dpc, Figure 1A). Embryos were collected once all rhombomeric boundaries are morphologically visible (9.5 dpc onwards). Out of 340 analysed embryos, 99 embryos showed recombination events within the hindbrain (F = 0.291). This analysis revealed an extensive population of clones that abutted but did not cross rhombomeric boundaries (34/116) (Table 2, Figure 1B). These clones displayed a clear tendency to expand within the boundary along the mediolateral (ML) axis (see white arrows in flat mounted-hindbrains of Figure 1B); indeed, all clones that respected boundaries displayed a larger ML than AP length. Many clones were located in the middle of the AP axis of the rhombomere, not reaching any of the boundaries (n = 70/116, see asterisks in Figure 1B). By contrast, very few clones contained cells located on both sides of the boundary, the clone having apparently crossed the boundary to spread in the adjoining rhombomere (n = 12/116, black arrow in Figure 1B). Most of the clones meeting a boundary, however, also respect it.

**Table 2:** Behaviour of the clones in respect to the rhombomeric boundaries.

	Theoretical	Observed	Expected	χ^2^ Discrepancy
Boundary-respecting	0.22	34	25.5	2.8
Boundary-crossing	0.57	12	66.1	44.3
Middle-clones	0.21	70	24.4	85.5
Total		116	116	132.6

Theoretical probabilities of crossing, respecting and failing to meet the rhombomeric boundaries by chance were computed from the sizes of clones relative to the AP length of the rhombomere, accounting for the measure error, as in ^[8]^. We checked whether observed data do fit this theoretical probabilities using a classical χ^2^ goodness-of-fit test. Results showed that we can reject the null hypothesis that observed data could come from the theoretical model (p<10^−28^, df = 2).

Boundary-respecting clones were generated by cells marked at all stages of development we studied (from 5.5–8.5 dpc). Indeed, when induction takes place once boundary formation has already started (E8.5), none of the observed clones crossed the boundaries (n = 30, data not shown). The existence of clones that respected boundaries and were induced as early during embryonic development as 5.5 dpc, indicates that the restriction along AP axis could precede morphological boundary formation. Previous data from Fraser et al. [8] in chick by the means of LRD-cell labelling showed similar results. They suggest that hindbrain boundaries either act as physical barriers to cell movement, or represent immiscibility interfaces due to molecular affinity constrains. Interestingly, the majority of clones that abutted the boundary display a shape which appears to outline it, as though cells had spread along the transverse extent of the adjacent boundary. Our results in embryos where recombination was induced at inner cell mass stage (5.5 dpc) suggest that positional information established at very early stages of embryonic development plays a relevant role for cell allocation along the AP axis later during development. Alternatively, several rounds of division may be required for the descendant of an arbitrary positioned parent cell to reach a position where, after this lapse of time, a boundary has already formed. If this was the case we would expect some parent cells already positioned near the prospective boundary. In this instance, we should see less disperse clones, hence bigger, which we do not observe. Because of the nature of the labelling we are using, which only allows us to get the information of the induced clones at the final stage of observation, we cannot unequivocally discern between those possibilities. However, if the tracing of the cells could be done live, we could overcome this uncertainty. Maybe luciferase marked cells, which can be visualised *in utero*, instead of GFP/LacZ systems, could be used when the technology becomes available.

Nevertheless, we have performed a statistic analysis of the probabilities of the clones being at a given position that let us propose that borders do make a difference in the sorting of the cells, although the mechanisms involved are not known. The probability that the pattern of distribution we observe could arise by random organization of the cells, assuming that there is no boundary restriction, is negligible (p<0.000001; Table 2).

### Statistical analysis supports that hindbrain boundaries behave as borders of cell restriction

**Figure 2:**
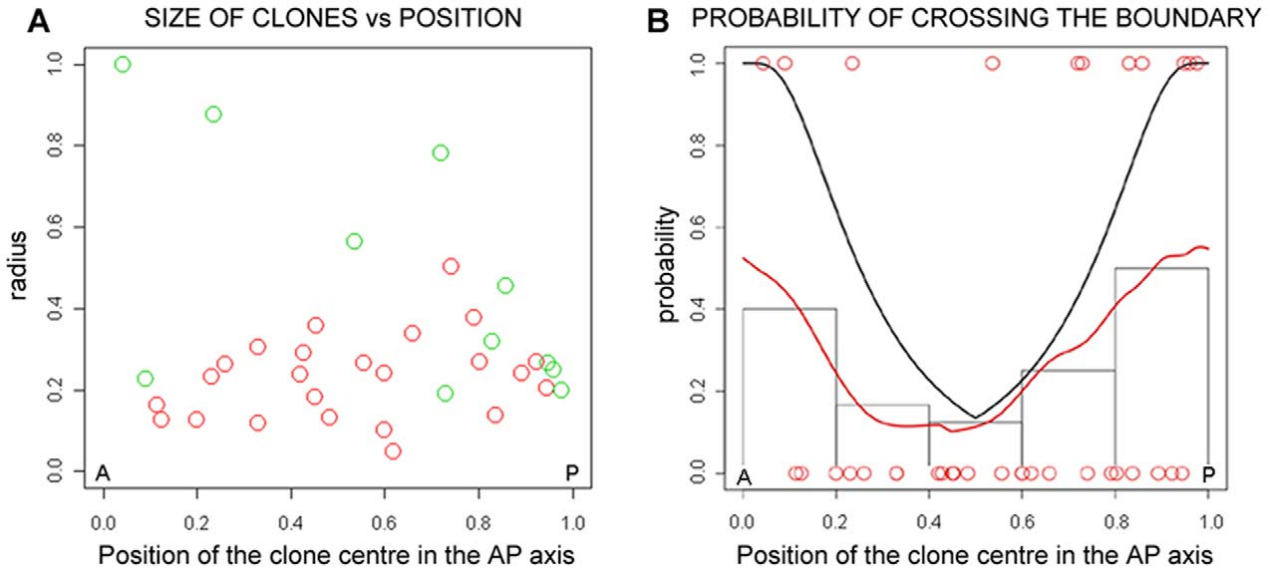
Estimated and theoretical probabilities for a clone to cross the boundary as a function of its position along the AP axis. (A) The scatter plot shows the size of the observed clones versus its centre position along the AP axis of the rhombomere. Radii have been measured as relative to the AP length of the rhombomere (see Materials and Methods). Colour of each clone has been chosen to reflect if it respects the boundary (red circles) or it crosses the boundary (green circles). (B) Estimated and theoretical probabilities for a clone to cross the boundary as a function of its position along the AP axis. Red circles show the horizontal position of the centre of observed clones along the AP axis of the rhombomere (it has been rescaled to [0,1]). Clones crossing the boundary have been placed in vertical position 1, clones respecting the boundary in vertical position 0. The red line is the probability of crossing the boundary as given by a generalized additive model fitted to data. Grey step lines show a discrete version of the estimated probability as given by the fraction of clones contained in each interval that cross the boundary. The black line is the probability of crossing the boundary as given by the theoretical model that assumes no boundary effect on the behaviour of clones.

To investigate the behaviour of boundary-respecting clones we generated a spatial model using mathematical tools. Since our technique does not allow targeting recombination to specific embryonic territories and axial levels, the average frequency of recombination for a particular rhombomere is quite low. To have a significant sample size we pooled all clones as belonging to a unique rhombomere that would extend along the AP axis. Only those clones between two recognizable boundaries were taken into account. r3 and r5 first appear as a very small band of cells, which expand in the AP axis over time, with the consequence that clonal dispersal in these rhombomeres might be different. However, due to the low frequency of recombination in single specific rhombomeres we had to dismiss this fact to obtain a significative sample of clones. First, we checked whether the position and size of a clone were independent. For that purpose, we measured the span of the clones (hereinafter called radius: the half-AP length of the clone relative to the rhombomere AP length) as a measure of the size (see Materials and Methods) and plotted the clones along the AP axis taking into account their size versus their position. Most clones have a radius between 0.1 and 0.7 (average radius = 0.30), with only 3 clones with a radius above 0.7 (Figure 2A). We found that clones of different sizes were equally distributed along the AP axis of the rhombomere (Figure 2A), strongly supporting the hypothesis that position and size were independent (see Materials and Methods).

Second, we estimated the probability for a clone to cross a boundary given the position of the clone centre along the AP axis (Figure 2B). The AP position was fixed as mentioned in Materials and Methods, considering all clones in a single unidimensional rhombomere, where anterior and posterior boundaries were identified. Clones (n = 35 red dots in Figure 2B) able to cross the boundary were placed in vertical position 1, while clones not crossing were placed in vertical position 0. The analysis showed that the probability for a clone to cross the boundary increased as its position along the AP axis was closer to the boundary (Figure 2B, red line and grey boxes).

In order to test whether the observed pattern could be generated by a random distribution (i.e. with no influence of the boundaries), we computed a theoretical probability function which represents a null-hypothesis, i.e. the probability for a hypothetical clone to cross the boundary if there was no influence of boundaries. This function was calculated from the distribution of the relative sizes of the clones according to their length in the AP axis (Figure 2B, black line, see Materials and Methods for more details). We observed that empirical data did not fit with the theoretical model. Although the probability for a clone to cross a boundary was higher when located close to that boundary, it was not as high as predicted by the null-hypothesis. This result suggests the existence of a boundary effect i.e., a restriction of cell movement due to the presence of the boundary. We propose this effect was affecting the cell behaviour before the morphological boundary was formed due to the timing of TM administration. However, as previously exposed, this observation could also be explained by the original position of the cell generating the clone being too far away from a border at the time of induction. Nevertheless, if that would be the case, we would expect bigger clones at the time of observation.

It is interesting to note an asymmetrical cell behaviour along the AP position within the rhombomere. As seen in Figure 2B, the clones that fit the model the least, and hence show a greater boundary effect, are the ones at the anterior part of the rhombomere. This can reflect a time delay in the establishment of the A and P rhombomeric boundaries, or/and can be due to differential positional information along the AP axis.

Although our results with embryos induced at 5.5 dpc (before boundaries are established) suggest that positional information plays a more important role that the previously conferred, we do not know the cellular mechanisms governing the formation of interhombomeric boundaries. One possibility is that rhombomeric cells share specific surface properties that render them immiscible with those of adjoining rhombomeres. Indeed, several studies support this hypothesis. Aggregation cultures of isolated rhombomere cells suggest the segregation of rhombomeres by differential chemoaffinity [14]. Experiments in zebrafish showed that the cell sorting between odd- and even-rhombomeres is mediated by differential expression of Eph receptors and ephrins [15]. Later results point to a segregation of cells from adjacent rhombomeres due to differential cell affinity through Eph and ephrins [16]. Another possibility is that specialized cells at boundaries generate a mechanical barrier to cell mixing. Evidences show that morphological boundaries are defined by unique molecular expression and functional properties, and boundary cells have an essential role in the control of neurogenesis [17].

With this analysis we show that in the mouse hindbrain, as in the chick's, rhombomeric boundaries are borders of cell restriction, and probably this effect happens much before the appearance of the molecular and morphological boundaries.

## Materials and Methods

### Mouse strains and treatment

The R26R-RERT or R26R-EYFP-RERT mice lines were used for clonal analysis; these are crosses between lines carrying the R26R [11] or R26R-EYFP reporter [12] and RERTn [18] transgenes. Labelled cells are produced by the activity of CreERT2, an inducible recombinase activated by 4-hydroxy-tamoxifen (TM). Ubiquitous CreERT2 expression is provided by a knock-in insertion of the CreERT2 cDNA into the 3′ UTR of the RNA polymerase II gene, which yields viable homozygotes with no obvious phenotype [10].

To produce experimental litters, double-homozygous RERT-EYFP males ^[10]^ were mated with C57Bl/6J or CD1 females. All resulting embryos were therefore double-heterozygous for the inducer (RERTn; [18] and reporter (R26R-EYFP; [12] alleles. For inducing recombination in these embryos, pregnant females from timed matings were injected intraperitoneally with 200 microliters of 4-hydroxytamoxifen (Sigma H6278) dissolved in corn oil (Sigma C8267) at the desired concentration (eg: a 0.25 mg/ml concentration for a 50-microgram dose).

Recombination events are highly dependent on tamoxifen administration; previously, the background (recombination events without induction) was determined to be very low and those events detected were predicted to occur at late stages in development [10].

All animals were handled in strict accordance with good animal practice as defined by the procedure used (CEEA JMC-07-1001-CPC), which has been approved by the institutional animal care and use ethic committee (PRBB-IACUC). Our Animal Facility, in accordance with national and European regulations, is registered as animal research center with the number B9900073.

### β-Galactosidase staining and immunostaining

For X-Gal staining embryos were collected at desired stages (9.5–10.5 dpc) and briefly fixed in 4% PFA. Staining was performed as described in [10].

For immunostaining, embryos were collected at desired stages (9.5–10.5 dpc) and fixed in 4% PFA. After dehydratation in methanol series they were stored at −20°C. Immunostaining was performed as described in [19]. Briefly, embryos were rehydrated and incubated with polyclonal antibody anti-GFP [1∶500] (Molecular Probes) overnight at 4°C. As secondary antibody goat anti-rabbit conjugated with alkaline phosphatase or horseradish peroxidase [1∶400] (Dako) were used. Embryos were washed and staining revealed by NBT-BCIP or DAB. Embryos were postfixed overnight in 4%PFA and stored in 50% glycerol. Hindbrains were dissected and flat-mounted. Pictures were taken under a Leica MZFLIII microscope with Leica DFC 300FX camera.

### Statistical analysis

As a first approach, to calculate the probability of the clones to cross the rhombomeric boundaries we applied the same rational than Fraser et al. [8]. They argue that, given the average spatial extent of the clones and the error margin in the measure, the theoretical probabilities of crossing, respecting and failing to meet the rhombomere boundaries by chance should be 0.4, 0.3 and 0.3 respectively. With the spatial extent of our clones and rhombomeres, and adjusting for the measure error, we estimate these probabilities to be 0.56, 0.22 and 0.21 respectively. To compare the expected frequencies under this model to the observed frequencies (see Table 2), we performed a chi-square test (df = 2) obtaining a very small p-value (p<0.000001).

### Clonal analysis

**Figure 3:**
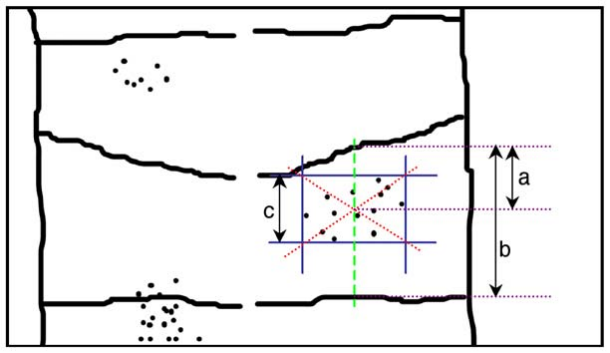
Measure of the clone size and position. Clone radius was measured as the half-AP length of the clone (c/2) relative to the rhombomere AP length (b), (radius = (c/2)/b. Position of the clone was established by measuring from the centre of the clone to its immediately anterior rhombomeric boundary (a). Position of the clone was then referred as a percentage of the length of the rhombomere at the level of the clone centre (b) (a/b x 100).

Cell clones were identified by *LacZ* or GFP staining. Individual clones were numbered and measured. Only those clones between two recognizable boundaries were taken into account to calculate the probabilities of boundary crossing. Clone radius was measured as the half-AP length of the clone (c/2) relative to the rhombomere AP length (b), (radius = (c/2)/b), and it was used as a measure of clon size. Position of the clone was established by measuring from the centre of the clone to its immediately anterior rhombomeric boundary (a). Position of the clone was then referred as a percentage of the length of the rhombomere at the level of the clone centre (b) (a/b x 100) (Figure 3).

### A simple spatial model

To achieve a more informational model we used the clone information in the form “position of the clone centre as % of AP length of the rhombomere” to place a clone in this interval. Thus, the rhombomere is the unidimensional space [0,1], being 0 anterior and 1 posterior. Note that since all clones have been placed in this single idealised rhombomere, our boundaries are both a posterior boundary of a rhombomere and the anterior boundary of the next rhombomere. This results in a circular model for estimation. The size of a clone was measured through its radius as a percentage of AP length of the rhombomere. Using this data we fitted a generalized additive model (binomial family) of the form
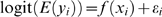



where *y_i_* is the binary response (1 being the clone crosses the boundary) and *x_i_* is the position of the clone centre. The fitted function is shown as the red line in Figure 2B. The model was fitted using the gam function in the package gam for the R statistical software [20]–[22].

### Theoretical Model

To check whether the boundary has an effect on the behaviour of the clones, we built a theoretical model (null-hypothesis) based on the following assumptions. (1) The position of the clone centre along the AP axis within the rhombomere is uniformly random along the [0,1] interval. The radius of the clone is (2) independent from its position and (3) distributed as a lognormal variable with parameters µ and σ, therefore log *R* is distributed as a normal. Let *L*(*x*) be the probability that a clone located at AP position *x* crosses the boundary and let *R* be its (random) radius. Then, for *x* in [0, ½],
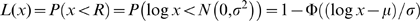



Where Φ() is the cumulative distribution of a standard normal (natural logarithms are used). For *x* in [½, 1], take *L*(*x*) = *L*(1–*x*). This function is displayed as the black line in Figure 2B. Parameters used were −1.34 and 0.64, the mean and standard deviation of the log of the observed radii.

The mentioned assumptions are supported by our data (see also Figure 2A). A Kolmogorov-Smirnov test for the uniform distribution of the clone centres along [0,1] gives a *p*-value equal to 0.51. The same kind of test applied to check the lognormal distribution of the clone radii gives *p* = 0.72. Furthermore, the fact that the growth of a clone is geometrical in time also supports this kind of distribution for its size. Independence between size and position is supported by the low correlation observed (*r* = −0.13) in our data (see also Figure 2A).
